# Supplementary material for: β-Glucosidase activity and antimicrobial properties of potentially probiotic autochthonous lactic cultures
Source: PeerJ. 2023 Oct 6;11:e16094. doi: 10.7717/peerj.16094 (PMC10561641; doi:10.7717/peerj.16094)
Supplement: Supplemental Information 1 [file peerj-11-16094-s001.docx]

**Determination of β-glucosidase activity**

The native strains, available in freeze-dried form, were grown in tubes containing modified De Man Rogosa e Sharpe (MRS) broth, prepared with peptone (manufactured by Laboratório Conda S. A., Spain, distributed by Kasvi, São José dos Pinhais, Brazil, 10 g), yeast extract (Himedia, Mumbai, India, 4 g), meat extract (manufactured by Laboratório Conda S. A., Spain, distributed by Kasvi, São José dos Pinhais, Brazil, 8 g), Tween (Dinâmica, São Paulo, Brazil, 1 g), monobasic potassium phosphate (Dinâmica, São Paulo, Brazil, 2.4 g), ammonium acetate (Vetec, Rio de Janeiro, Brazil, 2 g), magnesium sulfate (Sol-Tech, São Paulo, Brazil, 0.2 g), manganese sulfate (Vetec, Rio de Janeiro, Brazil, 0.0379 g) and distilled water (1 L), added cellobiosis (Scientific Exodus, São Paulo, Brazil, 20 g), incubated in a bacteriological stove at 37 °C for 24 h (first activation). Soon after, a new culture was performed transferring 100 μL of the first activation to glass test tubes containing 5 mL of modified MRS broth, incubated under the same conditions (second activation). The cultures were centrifuged for 15 minutes at 3000 rpm. The supernatant was used to determine the extracellular β-glucosidase activity and the cultures were resuspended in sodium citrate (50 mM, pH 4.8). Then, the cultures in the sodium citrate solution were sonicated (Unique, model Ultrasonic Cleaner 2500, Indaiatuba, Brazil) in steps with intervals of 5 s (equipment on) followed by 5 s of waiting (equipment off) totaling 40 min for enzyme release. Aliquots of 1 mL of each cell-free extract were incubated in 1 mL of cellobiose for 30 min at 50 °C (reaction mixture), interrupting the reaction by immersion of the tubes in boiling water for 5 min. The tubes were transferred to a bath in cold water and the glucose concentration was determined based on the glucose oxidase-peroxidase reaction. For negative control, time zero was used by adding 1 mL of the inactivated enzyme for 20 min in boiling water in tubes already containing 1 mL of cellobiose acclimatized to 50 °C. To determine the glucose concentration, 15 μL of the reaction mixture were pipetted into a tube containing 1.5 mL of the glucose oxidase-peroxidase reagent (GOD-POD, Biotécnica, Minas Gerais, Brazil), allowed to react for 10 minutes at room temperature, and the readings were performed in a spectrophotometer SP-2000 (Spectrum, Shanghai, China) at 500 nm. To zero the equipment, 1 mL of the GOD-POD reagent was used. The enzymatic activity was expressed in IU (μmol of product released per minute based on equation 1:

$$EA= \frac{D\times Concentration \left( \mu mol/mL \right)\times total dilution of the reaction mixture (ml)}{time (minutes)} (1)$$

Where:

*EA* = value of activity found (µmol/min)

Concentration (µmol/mL) of the unknown sample (X) = Y± b/a

Y = absorbance

a = angular coefficient of the line

b = linear coefficient of the line

*D* = enzyme dilution (if necessary dilute), if the glucose concentration obtained exceeds the linearity limit of the curve

Total dilution of the reaction mixture = 2

The determination of the enzymatic concentration expressed in U/mL was calculated by equation 2:

$$\left[ ENZ \right]=\frac{D\times Concentration \left( \mu mol/mL \right)\times total dilution of the reaction mixture\left( mL \right)}{time \left( min \right) \times supernatant vol \left( mL \right)}(2)$$

[*ENZ*] = concentration enzyme (µmol/min mL or U/mL)

Concentration (µmol/mL) of the unknown sample $\left( X \right)=Y\pm b/a$

Y = absorbance found

a = angular coefficient of the line

b = linear coefficient of the line

*D* = enzyme dilution (if necessary dilute), if the glucose concentration obtained exceeds the linearity limit of the curve

Total dilution of the reaction mixture = 2

To perform the standard curve, a standard solution of 10 µmol of glucose per mL was prepared and from it the following concentrations: 1, 2, 3, 4, 5, 6, 7, 8, and 9 µmol of glucose per mL were obtained. The glucose concentration was determined. The absorbance values ​​obtained were plotted in a graph, being absorbance (ordinate) × concentration in µmol/ mL (abscissa). The straight-line equation was used to calculate the enzymatic concentration and enzymatic activity of β-glucosidase.
