# Supplementary material for: β-Glucosidase activity and antimicrobial properties of potentially probiotic autochthonous lactic cultures
Source: PeerJ. 2023 Oct 6;11:e16094. doi: 10.7717/peerj.16094 (PMC10561641; doi:10.7717/peerj.16094)
Supplement: Supplemental Information 3 — Columns: (A) Salmonella typhimurium ATCC 14028 (I and II); (B) Staphylococcus aureus ATCC 25923 (III and IV); (C) Escherichia coli ATCC 25922 (V and VI). Wells: Lactiplantibacillus plantarum CNPC001 (1 and 10); Lactiplantibacillus plantarum CNPC002 (2); Lactiplantibacillus plantarum CNPC003 (3); Lactiplantibacillus plantarum CNPC004 (4); Limosilactobacillus mucosae CNPC007 (5); Lactiplantibacillus plantarum CNPC020 (6); Lacticaseibacillus rhamnosus EM1107 (7); Positive control (liquid) with 5 μg ciprofloxacin in solution (8); Negative control (9). Disc: Positive control with 5 μg ciprofloxacin (11). [file peerj-11-16094-s003.docx]

Figure S1. *In vitro* antimicrobial activity of potentially probiotic lactic acid bacteria strains against different pathogens.

| ***Salmonella typhimurium* ATCC 14028 (A)** | ***Staphylococcus aureus* ATCC 25923 (B)** | ***Escherichia coli* ATCC 25922 (C)** |
| --- | --- | --- |
| 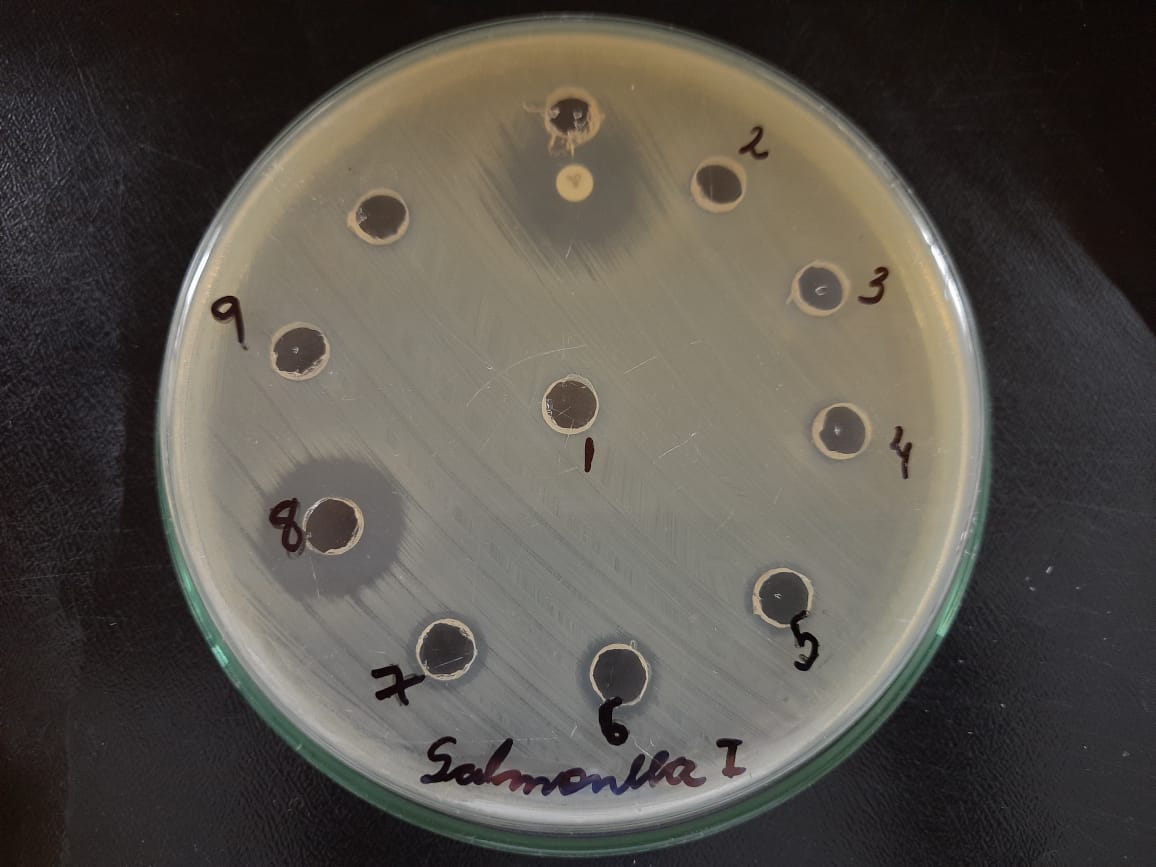  I)  10  11 | 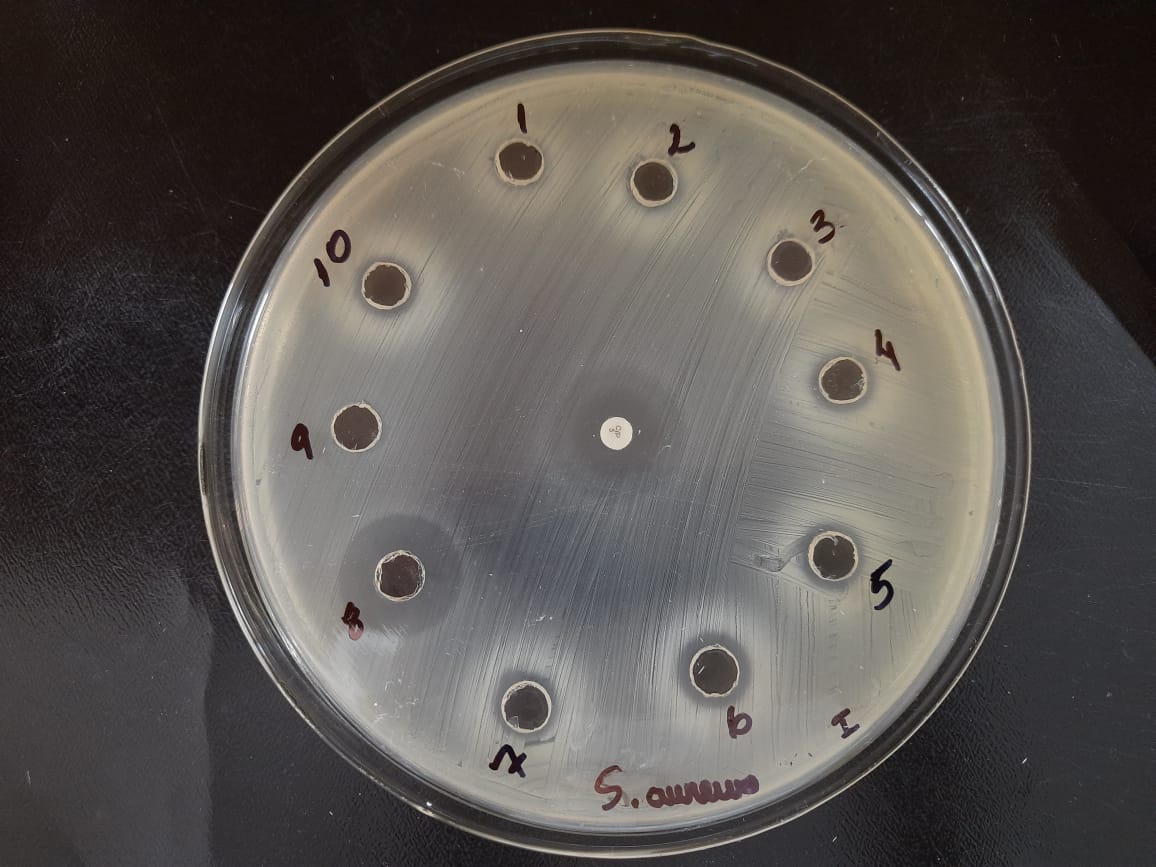  III)  11 | **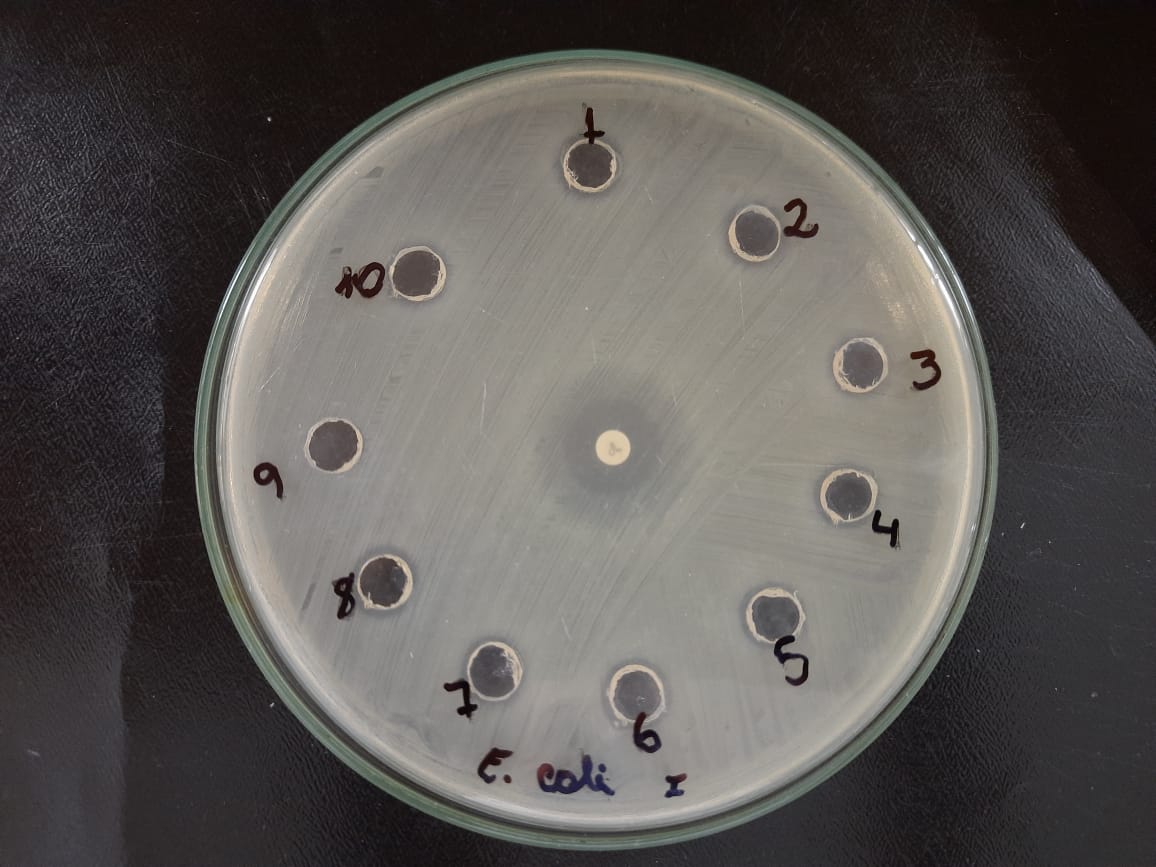**  V)  11 |
| 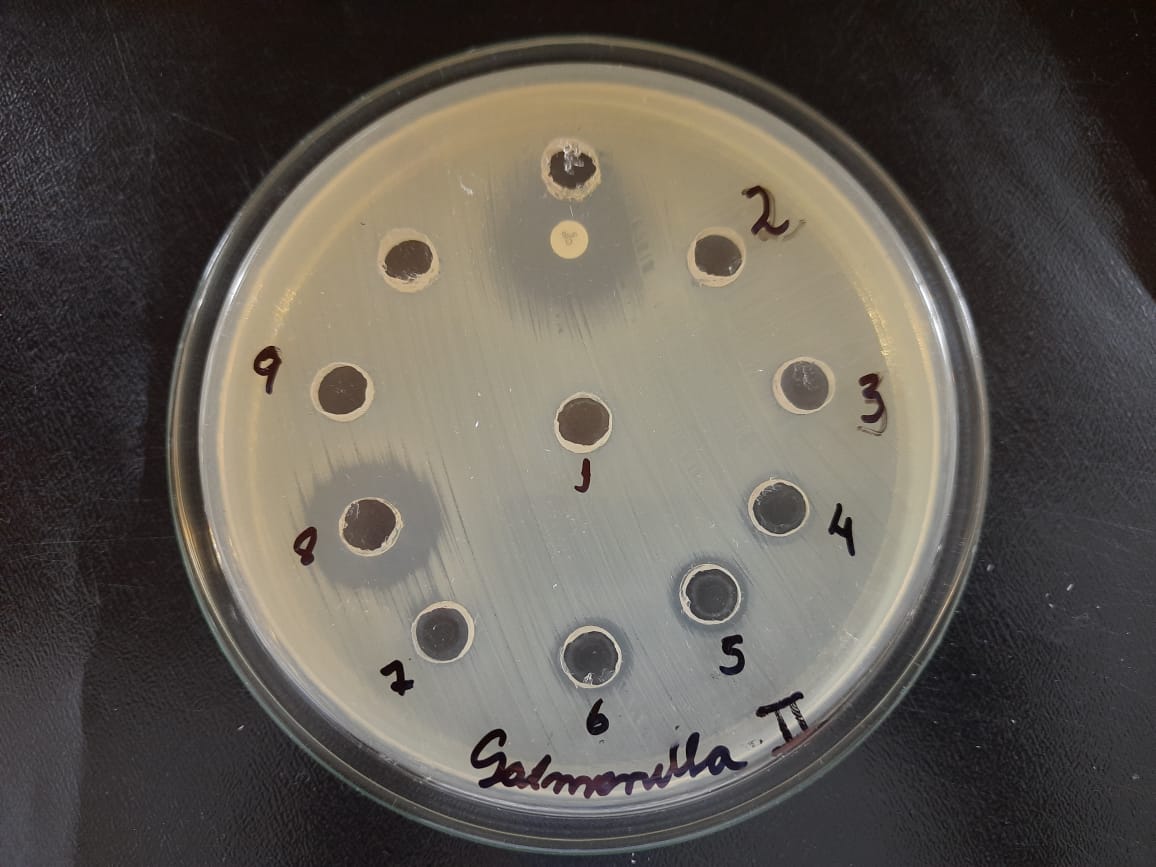  II)  11  10 | **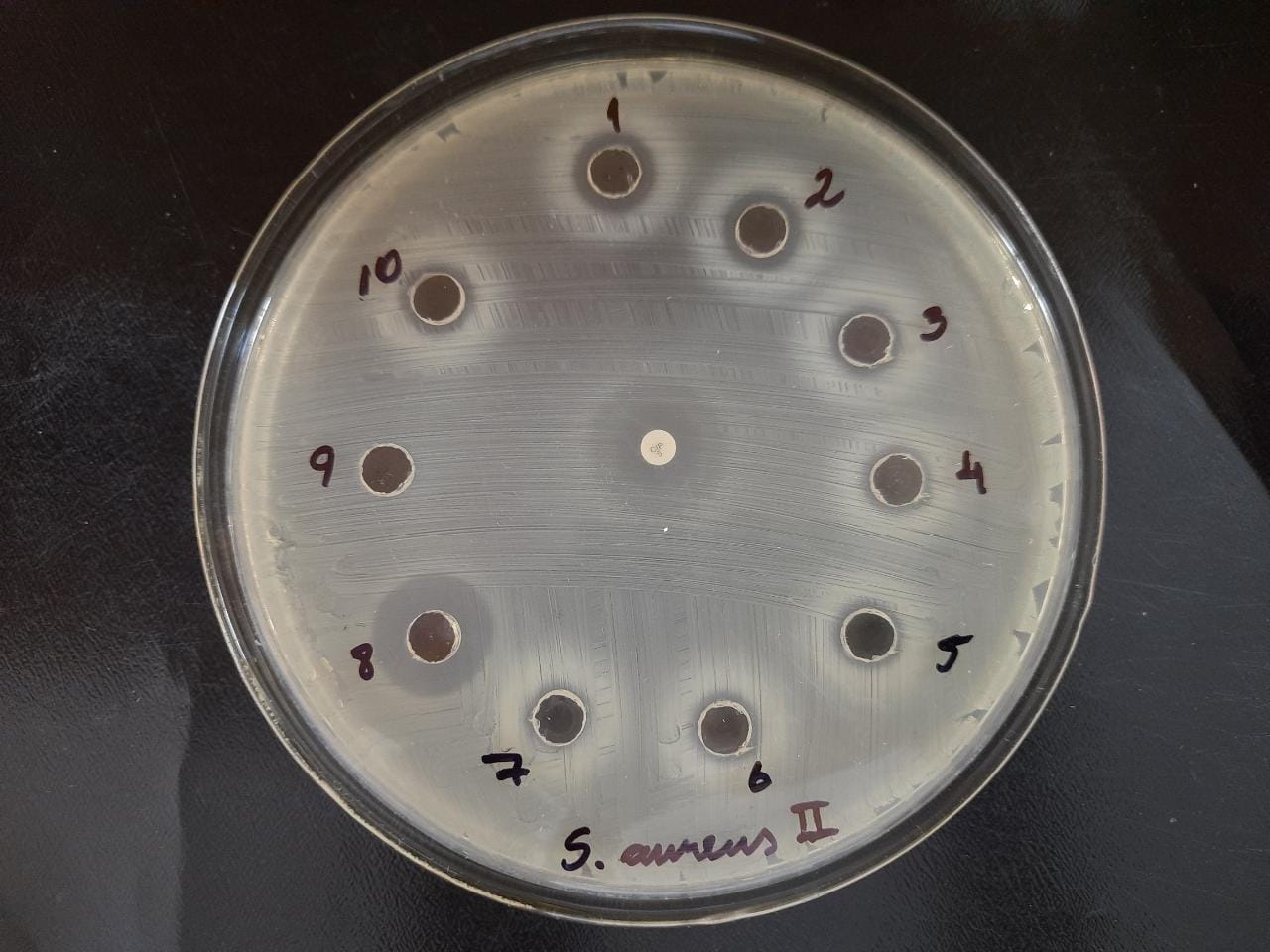**  IV)  11 | 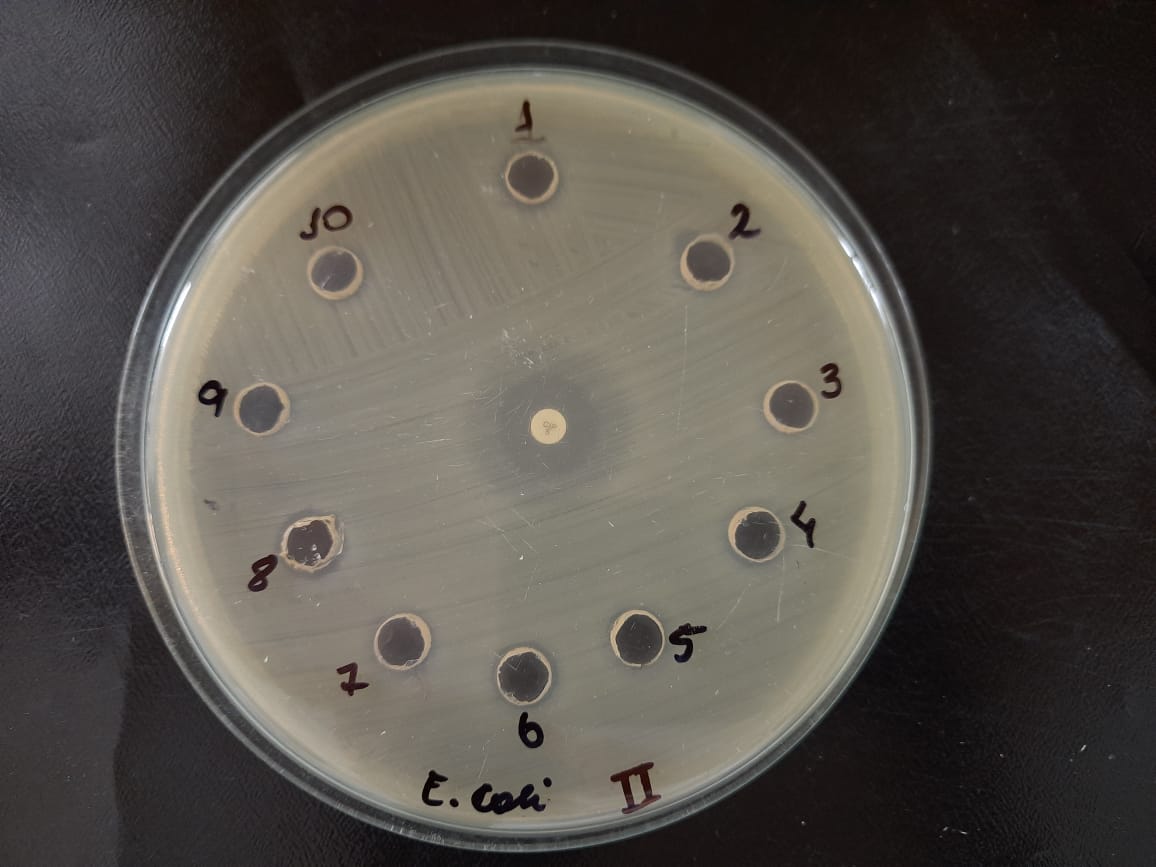  VI)  11 |

Columns: A - *Salmonella typhimurium* ATCC 14028 (I and II); B - *Staphylococcus aureus* ATCC 25923 (III and IV); C - *Escherichia coli* ATCC 25922 (V and VI)*.* Wells: *Lactiplantibacillus plantarum* CNPC001 (1 and 10); *Lactiplantibacillus plantarum* CNPC002 (2); *Lactiplantibacillus plantarum* CNPC003 (3); *Lactiplantibacillus plantarum* CNPC004 (4); *Limosilactobacillus mucosae* CNPC007 (5); *Lactiplantibacillus plantarum* CNPC020 (6); *Lacticaseibacillus rhamnosus* EM1107 (7); Positive control (liquid) with 5 μg ciprofloxacin in solution (8); Negative control (9). Disc: Positive control with 5 μg ciprofloxacin (11).
